# Non-intubated general anesthesia based on Bi-spectral index monitoring: Case reports of 2 patients undergoing endo-bronchial ultrasound guided trans-bronchial needle aspiration

**DOI:** 10.1097/MD.0000000000022458

**Published:** 2020-10-02

**Authors:** Xiaoxia Li, Changaramkumarath Gichin, Silin Xiang, Ling Zhou, Ling Chang

**Affiliations:** Department of Anesthesiology, The Second Affiliated Hospital of Chongqing Medical University, Chongqing, China.

**Keywords:** airway management, anesthesia recovery period, balanced anesthesia

## Abstract

**Rationale:**

Endo-bronchial ultrasound guided trans-bronchial needle aspiration (EBUS-TBNA) has been widely accepted as a safe and efficient technique for diagnosing patients with mediastinal/hilar lymphadenopathy and suspected cases of lung cancer. An effective anesthetic technique should provide comfort and quick recovery of patients while allowing the clinicians to obtain adequate tissue sample. Therefore we combined mask ventilation support (SIMV), BIS monitoring, and short-acting medication to achieve the effect mentioned above.

**Patient concerns:**

In this report, both patients had lung mass accompanied by cough that lasted for >2 weeks, and were admitted to hospital for further diagnosis and treatment to clarify the nature of the mass. To make a definite diagnosis, EBUS-TBNA examination was performed under general anesthesia. Both patients had no salient past history.

**Diagnosis:**

Case 1 was diagnosed as tumor or pneumonia based on the right lung shadow. Case 2 was diagnosed with squamous cell carcinoma of the right lung with right hilar lymph node metastasis. The diagnostic results of both patients were based on pathological examination of tissues obtained by EBUS-TBNA, of which case 1 required further confirmation by lung biopsy.

**Intervention:**

Both the patients received antibiotic treatment before EBUS-TBNA. We used the mask ventilation supported by SIMV mode without using muscle relaxant, thus providing a guarantee for rapid and high-quality recovery of patients.

**Outcomes:**

During EBUS-TBNA, the vital signs of the 2 patients were stable. Both patients recovered within 5 minutes after we stopped pumping general anesthetics. None of the patient complained of any discomfort and felt comfortable. No complications occurred during and 3 months after EBUS-TBNA examination.

**Lessons:**

The obtained results showed that this anesthesia scheme can provide appropriate depth of anesthesia for patients undergoing EBUS-TBNA examination, while ensuring rapid and high-quality recovery of patients.

## 1 Introduction

Endo-bronchial ultrasound guided trans-bronchial needle aspiration (EBUS-TBNA) was developed in 2002. Since 2007, EBUS-TBNA has been widely accepted by clinicians as a safe and efficient technique for diagnosing patients with mediastinal/hilar lymphadenopathy and suspected cases of lung cancer.[^1^,^2^,^3^]

Inputting the keyword “EBUS-TBNA” to search in PubMed generates 1002 related research documents, while imputing “EBUS-TBNA+GA (general anesthesia)” produces 7 related research documents. Among these 7 studies, 1 addresses the complications during EBUS-TBNA^[2]^; 2 focus on the impact of different TBNA methods on diagnosis[^2^,^3^]; 4 remaining articles include a study from 2010 dealing with the use of pethidine for EBUS-TBNA,[^4^,^5^,^6^,^7^] and other 3 papers that focus on the comparative study of GA and MS (moderate sedation) or deep sedation (DS).[^5^,^6^,^8^] The above results show that there are many methods available for EBUS-TBNA, including moderate sedation, deep sedation, general anesthesia, and monitoring anesthesia. At the same time, the influence of various anesthesia methods on diagnostic results is also lacking of large data support. As far as safety is concerned, general anesthesia is not hampered by the decrease of oxygen saturation due to the support of mechanical ventilation. As far as recovery speed and quality are concerned, moderate sedation and monitoring anesthesia can ensure rapid and efficient recovery by retaining the patient's spontaneous breathing. Currently, there is no such approach that could simultaneously ensure the quick and efficient oxygen and recovery of patients. Consequently, the aim of this study was to explore an anesthesia strategy EBUS-TBNA, which can combine the advantages of GA and MS, and overcome their respective shortcomings. For example, the use of GA muscle relaxants can delay recovery, while MS may cause intraoperative cough and affect the collection of specimens, thus affecting the diagnostic results to a certain extent. For these reasons, we chose the mask ventilation GA supported by SIMV instead of tracheal intubation GA, which effectively solved the recovery delay caused by muscle relaxant residues and ensured the oxygenation in the operation. On the other hand, we used Bi-spectral index (BIS) monitoring throughout the whole process, which fundamentally ensures that the whole examination process maintains uniform, stable, and appropriate depth of anesthesia. Thirdly, the combination of drugs with short half-life can ensure that once the drug is suspended, the patient can recover quickly, almost without any drug residues.

## 2 Case presentation

### 2.1 Case 1

**Figure 1:**
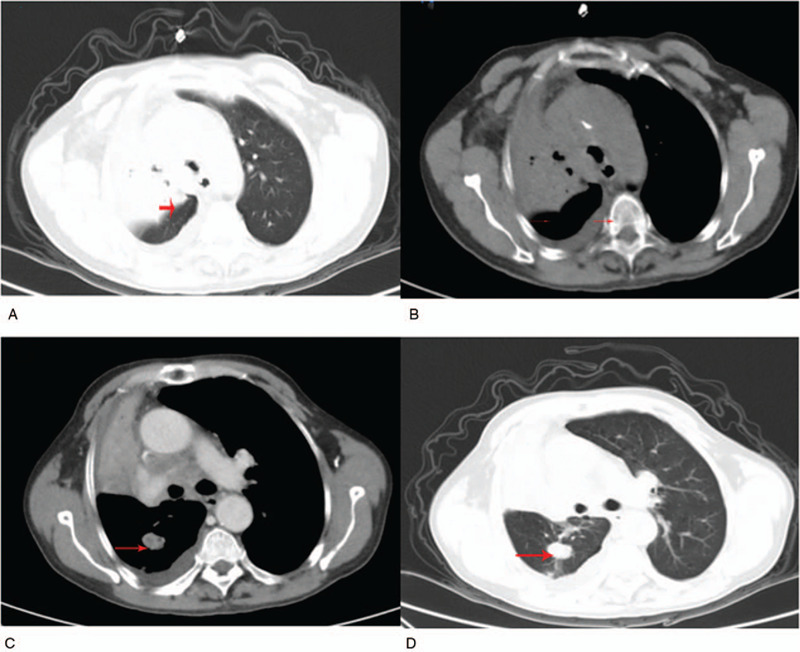
Computed tomography (CT): (A–D—axial CT images) results showed multiple patchy opacities in the anterior segment of the right upper lobe, left lower lobe, and lingual segment of left upper lobe along with multiple para-mediastinal and right hilar lymph nodal enlargements.

A 61-year-old female Han patient was admitted to the respiratory department of the hospital on March 5, 2019, where she was diagnosed with “lung shadow” because of coughing and sputum that were present for more than a month. Since onset, her appetite was good as well as her spiritedness and physical energy. Defecation and urination were normal; her body weight reduced (details unclear). Physical examination revealed rough breath sound. Chest radiographs showed increased density in the right lung, increased and blurred porta pulmonale, and suspicious mass. Aortic sclerosis. CT findings suggested that differential diagnoses included segmental pneumonia and lung cancer (Fig. 1). Consequently, EBUS-TBNA was indicated for further diagnosis. EBUS-TBNA lasted for 45 minutes. It took a total of 50 minutes for the patient from induction of anesthesia to recovery. During EBUS-TBNA, the patient's blood pressure was maintained at 85 to 146/50 to 80 mmHg, and the BIS fluctuated between 41 and 57 (Table 1). The results showed that protuberance and dorsal right lower lobe were chronically inflamed; R10 revealed granulomatous inflammation, and inflammatory cells were found in fibrous tissue punctured by R11 and R12. Cytological reports of brush tissue suggested necrotizing substances, some bronchial epithelial cells and inflammatory cells. The results of biopsy showed inflammatory changes.

**Table 1:**
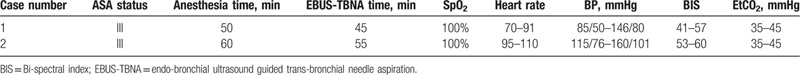
Vital signs.

### 2.2 Case2

**Figure 2:**
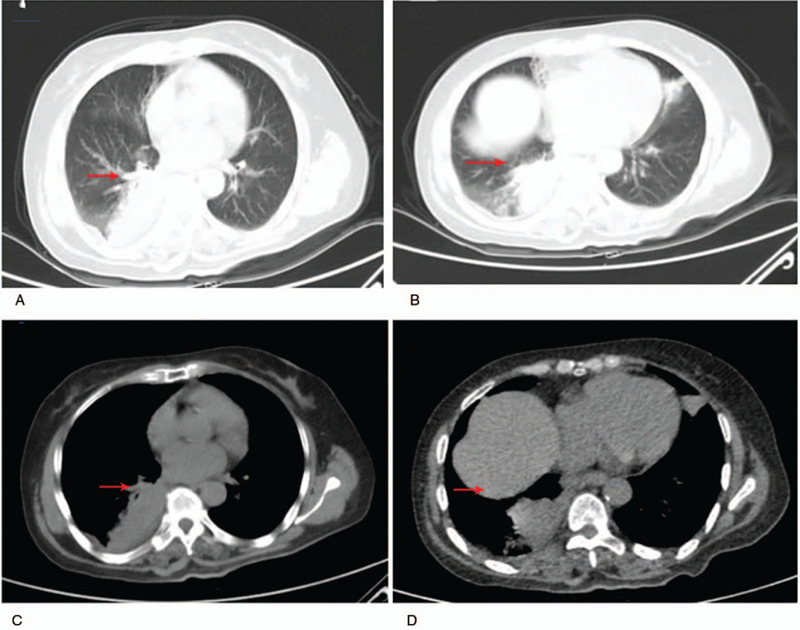
CT (A–D—axial CT images) results showed bilaterally emphysematous lungs with low density para-mediastinal mass in the right upper lobe, obstruction of right upper and middle lobe bronchus with subsequent atelectasis. In addition, vascular invasion was observed in the right upper pulmonary arteries, pulmonary veins, azygous vein and superior vena cava. CT = computed tomography.

A 62-year-old male Han patient was admitted to the Department of Respiratory Medicine of the hospital on March 4, 2019 because of repeated coughing and sputum that lasted for more than 20 days. The patient felt dyspnea on exertion after physical activity, while his chest x-ray showed patchy shadow of the right upper middle lung, unclear right hilum, emphysema of both lungs, and increased texture of both lungs. There were a few fibrous foci in the left lower lung. Initial treatment (peracillin sodium, 5 g of sulbactam sodium powder administered through intravenous twice a day) with antibiotics showed no improvement. Computed tomography (CT) (Fig. 2) results suggested central lung cancer as possible diagnosis, and EBUS-TBNA was indicated for further investigation. EBUS-TBNA lasted 55 minutes. It took 60 minutes for the patient from induction of anesthesia to recovery. During EBUS-TBNA, the patient's blood pressure was maintained at 115–160/76–101 mmHg, and the BIS fluctuated between 53 and 60 (Table 1). EBUS histopathological examination revealed that right middle lobe non-small cell carcinoma might be squamous cell carcinoma, which needed to be further verified with immunohistochemical confirmation. Histological examination showed atypical cells, and squamous cell carcinoma was considered. The biopsy results were consistent with the above results.

### 2.3 Technique

Physical status of both patients was American Society of Anesthesiology (ASA) III. Drugs that were used can be seen in Table 2. Total intravenous general anesthesia was applied for EBUS-TBNA. Face mask ventilation (FM, Boyi Medical Devices Co., Ltd., Shanghai, China) was provided supported by Synchronized intermittent mandatory ventilation (SIMV) mode on the ventilator. SIMV allowed for synchronization of the ventilation of the patients’ breathing, thereby causing lesser stacking of breaths and air trapping. BIS was used to monitor the depth of anesthesia and maintained between 40 and 60. Pulse oximetry was attached to the patients’ right thumb to monitor capillary oxygen saturation (SpO_2_). Electrocardiogram (ECG) leads and blood pressure (BP) cuffs were also attached on both patients to monitor their hemodynamic stability during the procedure. End tidal carbon dioxide was also monitored during the procedure. The intraoperative monitoring results are shown in Table 1.

**Table 2:**
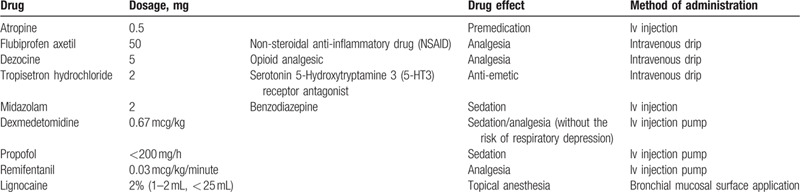
Drugs used.

Furthermore, the patients were observed for any drop of their baseline BP, cough reflex or movements during the insertion of the bronchoscope and throughout the procedure. A drop of >20% of patient's baseline BP was to be managed with 1 mg of Dopamine IV, while cough reflex/movements were to be managed with 1 mL of propofol IV; nonetheless, none of these were used since our patients did not exhibit either of these problems and remained hemodynamically stable throughout the procedure. After the procedure, the syringe pumps of propofol and remifentanil were stopped and flumazenil (0.5 mg), which is an antidote for midazolam, was intravenously administered and the patients were revived within about 5 minutes. The bronchoscopist obtained enough tissue samples from both patients. Histopathological examination in case 1 indicated chronic mucosal inflammation; in case 2, histopathological examination showed that non-small cell carcinoma tended to be squamous cell carcinoma, which required immunohistochemical analysis to confirm the diagnosis. The same physician (Associate Chief Physician with over 5 years’ work experience) attended both cases using the same instruments.

## 3 Discussion

Over the past decade, EBUS-TBNA has been seen as the first line diagnostic procedure for mediastinal and hilar lymphadenopathy.[^1^,^2^,^3^] Anesthetic considerations for the procedure have been an area of much interest and there are plenty of studies making comparisons between different methods.[^4^,^5^,^6^,^7^,^8^,^9^] EBUS-TBNA under GA as well as with moderate sedation (MS) seems to be safe,^[6]^ effective, and with good diagnostic yield but with comparable disadvantages and advantages, one of them being the higher cost.^[10]^

GA with neuro-muscular blocking agents (NMBA) provides a major advantage of having optimal conditions for the bronchoscopist, contributing to better diagnostic accuracy and optimal patient comfort. The disadvantages include establishing an artificial airway, mechanical ventilation, longer recovery, hemodynamic complications, and potentially higher cost. MS does not require the use of artificial airway, allows for quicker recovery, and has lesser hemodynamic impact with economic feasibility. MS, however, increases the incidence of cough and body movements and has variable levels of oxygen saturation.

Under our method of anesthesia, we administered intravenous GA without the use of NMBAs. This prevented the inhibition of spontaneous ventilation and paralysis of the diaphragm which allowed us to do face mask ventilation (FMV) on SIMV mode maintaining patients’ SpO_2_ at 100% throughout the procedure. In the current study, we used SIMV to manage the airways. SIMV is also called intermittent demand ventilation (IDV) on existing ventilators. In this mode the ventilator gives the patient instructional ventilation according to the preset breathing parameters (breathing frequency, tidal volume, breathing ratio, etc.) in every minute, and assists the patient to complete the spontaneous breathing when there is spontaneous breathing in the trigger window; if there is no spontaneous breathing in the trigger window, positive pressure ventilation in the gap is given at the end of the trigger window. Similar to assisted-controlled ventilation, SIMV allows patients to breathe spontaneously between 2 mechanical breaths. The main advantages of SIMV are reduced confrontation between spontaneous breathing and ventilator, reduced difficulty of weaning, reduced airway pressure, lack of respiratory muscular atrophy and dyskinesia, and reduced impact of breathing on cardiovascular system. In our cases, the ventilation parameters were as follows: volume tidal was 8 mL/kg, respiratory frequency was 12 times per minute, I/E was 1:1. The advantage of SIMV is that it allows adequate ventilation to patients regardless of their spontaneous respiration during the procedure. This is the reason why the pulse oxygen saturation in both of our cases during the procedure was 99% or 100%, and the end tidal carbon dioxide was kept between 35 and 45. SIMV provides the safety of airway during EBUS-TBNA.

In their previous studies, Fernandes et al^[6]^ and Casal et al^[8]^ compared the application of GA and MS in the process of EBUS-TBNA. They pointed out that because some pulmonary physicians did not accept GA, they turned to explore a suitable method for MS of EBUS-TBNA. Their results showed that although MS can provide approximately the same levels as GA, GA can provide better assurance because of the increased possibility of coughing, decreased comfort, and airway safety during the examination. Based on the previous research results, we chose GA to explore a more suitable method for EBUS-TBNA. We found that FMV combined with SIMV mode not only ensured adequate oxygenation, but also eliminated the use of muscle relaxants in traditional GA, and required only the use of short-acting anesthetics to ensure the high quality and rapid recovery of patients after the procedure.

Two of our cases were monitored by BIS, which ensured that EBUS-TBNA was provided with an appropriate depth of anesthesia and effectively prevented intraoperative awareness. Titration of the sedation level by BIS values can facilitate a quicker wake-up from general anesthesia and a shorter stay in the bronchoscopy room.

Furthermore, neither of our patients showed signs of hemodynamic instability despite their older age and did not exhibit cough reflexes or movements. No major drug-related adverse effects were observed in either of our patients.

The presented method also has some shortcomings, such as the non-protective airway of mask ventilation support, and the potential risk of regurgitation and aspiration. On the one hand, EBUS is an elective examination; patients need to fast, so the risk of aspiration is relatively small. On the other hand, once a large amount of active bleeding in the airway occurs over a short time, mask ventilation appears to be very passive, which is not as safe as tracheal tube.

Therefore, a comprehensive and correct assessment of the patient's condition is needed before the examination, and a full communication with the endoscopy doctor is needed in order to select the best regimen for the patient. The safety of anesthesia regimens should always be the primal consideration.

## 4 Conclusion

Mask ventilation combined with SIMV ventilation mode of general anesthesia can provide satisfactory anesthetic effects for EBUS-TBNA, as well as promote high-quality recovery of patients. The whole BIS monitoring ensures that the patient is in a moderate anesthetic depth. This approach is also suitable to avoid intraoperative awareness or too deep anesthesia. Rapid awakening of patients can improve the efficiency of the use of endoscopy room.

## Author contributions

**Conceptualization:** Xiaoxia Li.

**Data curation:** Silin Xiang, Ling Chang.

**Formal analysis:** Ling Zhou.

**Investigation:** Ling Zhou.

**Writing – original draft:** Changaramkumarath Gichin.
